# Conveying Sport Nutrition Information in YouTube Videos: A Qualitative Content Analysis of Dietary Advice and Ways of Communication

**DOI:** 10.1016/j.cdnut.2025.107525

**Published:** 2025-08-06

**Authors:** Anna Kiss, Orsolya Tompa, Zoltán Lakner, Brigitta Unger-Plasek, Ágoston Temesi, Sándor Soós

**Affiliations:** a Faculty of Education and Psychology, ELTE Eötvös Loránd University, Budapest, Hungary; b Department of Science Policy and Scientometrics, Library and Information Centre of the Hungarian Academy of Sciences, Budapest, Hungary; c Pro-Sharp Research and Innovation Centre, Budapest, Hungary; d Department of Agricultural Business and Economics, Institute of Agricultural and Food Economics, Hungarian University of Agriculture and Life Sciences, Budapest, Hungary; e Tashkent State Agrarian University, Tashkent, Uzbekistan

**Keywords:** qualitative content analysis, sport nutrition, social media, communication features, sport nutrition education

## Abstract

**Background:**

YouTube is one of the most widely-used social media platforms and has become a key source of nutritional information for athletes. Both experts and nonexperts use it as an educational tool; however, videos created by nonexperts are more popular among viewers. As social media sources can influence athletes' nutritional knowledge, it is essential that reliable nutritional information reaches them.

**Objectives:**

This study aimed to identify the key nutritional information and communication methods used in popular sports nutrition videos on YouTube.

**Methods:**

A systematic search was conducted on YouTube to select videos that met the following criteria: English language, sports nutrition-related content, available audio, free access, 4–20 min in length, and classified as informational or educational. Qualitative content analysis was performed to examine video content, and formal concept analysis was applied to determine the structure of associations among communication methods, sports nutrition themes, and presenter expertise. A total of 114 YouTube videos met the inclusion criteria.

**Results:**

Four themes emerged regarding sports nutrition messages: the function of nutrition in sports, know-how, dietary strategies, and developing a dietary framework. We identified four themes in the methods used to convey these messages: language features, content delivery methods, appearing connected to the audience, and establishing credibility. The analysis revealed distinct differences in communication approaches between experts and nonexperts. Expert videos often lacked the communication techniques that nonexperts used to build trust and connect with viewers.

**Conclusions:**

This study highlighted the key sports nutrition information and the characteristics of communication features in sports nutrition YouTube videos. The differences in communication methods between experts and nonexperts underscore the need for more effective strategies from experts to engage athletes and build trust. Collaboration between experts and nonexperts could help improve the quality and credibility of online content.

## Introduction

The Internet, especially social media, has become a dominant source of advice and information related to food and nutrition for the general population [1,2]. In addition to seeking professional guidance, athletes often use the Internet—including social media and other web-based technologies—as a source of nutritional information [3,4]. The vast amount of nutrition-related content available on social media platforms makes it challenging for athletes to discern between falsehoods and evidence-based recommendations and to determine reliable dietary choices for optimal performance and health.

YouTube has become one of the most popular social media and video-sharing platforms, with over 2 billion users and >500 h of video content uploaded every minute [5,6]. This free-access platform is commonly regarded by users as a source of medical and health-related information. Furthermore, consumers often base healthcare decisions on information acquired from watched videos [7]. Due to its large and diverse audience, YouTube has become a significant tool for science communication and patient education, helping people engage with and better understand various health topics. However, because of the open nature of the platform—where anyone can upload content regardless of their expertise—videos made by nonexperts are often of poor quality, misleading, or invalid [8]. Since 2007, studies have consistently found that health-related and medical information on YouTube is frequently biased and misleading [[9], [10], [11], [12], [13], [14]].

Social media is increasingly gaining acceptance as an educational platform among food and nutrition professionals [15], with influencers and athletes also using it to disseminate nutrition information [16]. Although experts are often cited as reliable sources, health videos created by nonprofessionals tend to be more popular among viewers. According to Langford and Loeb [17], videos that receive low ratings from experts tend to have higher view counts and better rankings on YouTube. Kiss et al. [18] found that the accuracy and quality of 114 YouTube videos on sports nutrition were low. Furthermore, their results suggest that YouTube users prefer videos rated as less reliable or of lower educational quality by experts.

Web-based sources can influence athletes’ knowledge of food, nutrition, and health, as well as affect their food choices [19]. Effective communication regarding sports nutrition can significantly shape athletes’ nutritional behaviors [20]. Therefore, athletes need access to reliable, evidence-based nutritional information. The content and style of sports nutrition videos on YouTube are particularly relevant, as the platform can be a valuable source of information for athletes. Studies on this topic can help us understand how nutrition professionals might better deliver effective dietary advice to athletes and positively influence their food choices.

Previous studies have primarily focused on the communication methods of healthy eating and nutrition messages on websites and print media [[21], [22], [23], [24]], with relatively few analyzing content on video-sharing platforms. Research evaluating dietary advice in blogs and magazines has revealed a strong pseudoscientific discourse and limited contextualization of emerging nutritional science [23,24]. Rogers et al. [16] found that social media influencers carefully select their content and communication strategies to ensure their nutritional messages resonate with users. The role of social media influencers in public health communication is gaining increasing attention [25], and Rogers et al. [16] emphasized that understanding how popular nutrition videos convey dietary information can help develop effective strategies to build trust in food and nutrition messages.

The dietary claims made in sports nutrition YouTube videos have not yet been systematically examined. This study aims to explore the main messages of popular sports nutrition videos and the ways in which nutrition information is communicated on YouTube. Our findings offer practical recommendations for sports nutrition experts on how to effectively convey evidence-based messages, build trust, and produce engaging and reliable sports nutrition content.

## Methods

### Overview

We used a mixed-methods approach, which involved conducting a quantitative study first [18] followed by a qualitative study [26,27]. Considering the broad and exploratory nature of the research aim, we adopted a general approach to qualitative research. A general qualitative approach is commonly used when there is limited knowledge about the topic under investigation and the research questions do not align with traditional methodological frameworks (e.g., case study, phenomenology, narrative research) [28,29]. Specifically, this study followed qualitative description [30], a widely applied method in the field of sports science [31]. Qualitative description aims to provide a comprehensive and descriptive understanding of a phenomenon (e.g., how sports nutrition messages are communicated by presenters in YouTube videos), with particular emphasis on offering insights for practitioners [30].

### Sampling and selection

We conducted a systematic search for videos containing relevant information about any aspect of sport nutrition on YouTube [32] on 10 January, 2023. We selected videos based on Fink and Mikesky’s [33] definition of sport nutrition, which encompasses not only elite athlete nutrition but also strategies for recovery after intense physical activity and overall health and wellness. To ensure the objectivity of the search results, we used incognito mode. To select relevant search terms, we used Google Trends [34]) to identify possible terms and estimate their relative search frequency. Our aim with this approach was to identify widely-used search terms related to sports nutrition content that would yield a specific sample frame. The terms “sport nutrition” and “exercise nutrition” yielded the most search results, whereas “athlete food and nutrition” and “athlete diet” also generated >1 million video records. After conducting the search using these 4 terms, we screened the first 4 pages of results on YouTube, with each page containing 25 videos per term. On the basis of previous research suggesting that users rarely browse beyond the third page of results [35], we limited our sample to the first 4 pages per search term. To select videos for analysis, we applied 2 filters: relevance (most relevant videos based on search terms) and duration (between 4 and 20 min). The 4–20 min range for video duration was chosen based on literature indicating that the optimal length for maintaining viewer engagement on YouTube typically falls between 7 and 15 min [36]. A lower threshold of 4 min was set to ensure that included videos contained sufficient content depth for meaningful analysis. Videos meeting the criteria outlined in Table 1 were analyzed. We retrieved video metadata using the Google YouTube API (2023) [37].

**TABLE 1:** Inclusion criteria for video selection.

Inclusion criteria
English language, sport nutrition-related content, available audio content, free access, videos between 4 and 20 min, informational or educational videos

### Data analysis

For video analysis, we applied qualitative content analysis, a commonly used and preferred method in qualitative description research [30]. This method has become increasingly prevalent in the examination of digital content, such as written and visual material on Internet forums, websites, and social media platforms [31,38]. A key feature of this method is its ability to explore both the manifest and latent content of texts. In our analysis, manifest content was examined to identify the main sports nutrition messages, whereas latent content analysis focused on how these messages were conveyed, revealing deeper meanings. Given the limited and fragmented prior knowledge about the phenomenon under investigation, we chose an inductive approach to data analysis [39]. We obtained video transcripts using Google’s automatic transcription service. Coding was performed by 2 researchers (AK and OT) using Atlas.ti software [40] as a qualitative data analysis tool. Both researchers hold formal university degrees in Nutrition and Dietetics, along with PhDs in Food Science.

The analysis of messages conveyed in sports nutrition videos followed the inductive process outlined by Cho and Lee [41], a 5-step process to guide the procedure and facilitate rigorous data integration. First, each video was assigned a unique identifier, and the transcripts were read and re-read to gain a deeper understanding of the content. Simultaneously, open coding was used to identify relevant concepts and initial thoughts related to the research question, and notes were taken. In this initial phase, video transcripts were reviewed line by line and annotated with descriptive labels to capture relevant concepts and units of meaning. After open coding, both researchers independently identified preliminary codes—an initial set of labels representing meaningful text segments—which served as the foundation for developing broader categories in the subsequent stages of the analysis. In the third step, the coded data were organized, and initial subcategories were created, followed by the development of main categories shaped by recurring themes in the text. In the fourth step, transcripts were re-read and the categories further analyzed, organizing them into higher-order, broader categories to form a comprehensive and coherent pattern. Finally, the 2 researchers compared and discussed their findings until they reached a consensus on the interpretation of the data. They resolved any discrepancies in code assignments by discussing the rationale behind each decision and agreeing on a final assignment. This process allowed them to fine-tune the codes and subcategories, leading to the identification of the final categories and themes. Final themes were developed through consensus involving all authors, ensuring they were exhaustive and relevant to the research question.

During the inductive analysis, the emerging thematic structure of how the messages were communicated aligned in part with the framework established by Rogers et al. [16]. As a result, we adopted their framework, where applicable, to our study, enabling the analysis to be seen as both inductive and deductive. This approach strengthens the validity of our findings, as the alignment between the 2 thematic structures provides further support for the results.

Quotes were chosen to illustrate both shared and differing perspectives of the video presenters. These quotes were included alongside the findings. It is important to note that the study did not analyze digital reactions from third-party individuals, such as viewer comments.

### Determination of expertise

In addition to identifying the key dietary information and how it was communicated in the videos, we also determined whether the video presenters could be considered experts. Individuals were classified as experts if they were either Registered Dietitians or held a Bachelor's or Master’s degree in Nutrition. This classification was established through a combination of self-disclosures made in the videos and independent verification. We cross-checked professional credentials using publicly available data from personal or professional websites, social media profiles, and other relevant sources. In the qualitative analysis, we coded videos by experts and nonexperts together, which allowed us to identify both shared and distinct themes and differences in what and how messages were conveyed across the 2 groups. Some categories, such as personalized nutrition, emerged in both groups but with differing content. Consequently, the results consistently indicate differences between the 2 groups, ensuring that such nuances were thoroughly captured. This approach offered a more comprehensive interpretation of the data.

## Formal Analysis of the Structure of Associations between the Communication, Video Theme, and Expertise

In this analysis, we examined the key dietary information, how this information was communicated, the expertise of presenters, and the structure in which this information was presented. To discern and comprehensively analyze the interrelations between these aspects of the sample, we adopted a formal methodology from the field of Knowledge Representation. We employed formal concept analysis (FCA), which enables the identification of associations and logical relations between coded features in a given topic [42]. In this case, FCA was applied to analyze communication methods, sports nutrition themes, and presenter expertise. Technically, the FCA procedure builds a concept lattice based on the co-occurrence of codes. A concept lattice is a specialized network composed of nodes called formal concepts [43]: in our case, these represent distinct associations among video codes emerging from the sample, along with the videos corresponding to those associations. A formal concept embodies an empirical category defined by codes and corresponding videos: the former (associated codes) is called the *intent* of the category—that is, the concept's meaning. The latter (videos) is called the *extent*—that is, the set of objects of analysis represented by the concept. The connections between formal concepts, or the edges of the network, represent a specific relation—namely, the subset relation. The resulting network thus forms a conceptual hierarchy or taxonomy of categories. Importantly, this structure allows for the inference of individual codes or code groups based on the presence of others; these deductive inferences are referred to as the *implication set* induced by the lattice. The implication set describes the most significant associations between the coded aspects of the videos, making this method well-suited to our analytical aims. The calculation of formal concepts and the visualization of the concept lattice were performed using ConExp software [44] and the R statistical software [45].

In this study, a formal concept lattice built from the incidence matrix of videos (objects of analysis) and features (categories) (Supplemental Table 1) is called a “formal context,” it is best analyzed in terms of implications. As mentioned above, implications or implication sets explicate the knowledge coded in a network of formal concepts. It should be noted that the full set of implications (deductive inferences) is algorithmically calculated from the structure of the concept lattice, that is, from concept—subconcept relations. A detailed description and visualization of the concept lattice are provided in Supplemental Figures 1 and 2 of Supplemental Material 1.

Implications define expert communication in terms of methods and main sports nutrition messages, in contrast to nonexpert communication. For a systematic analysis of the results (the full implication set), we focused on 2 subsets of implications that are directly related to our research aims regarding the relationship between expertise and communication style or main messages. Implications are also being ranked according to a score, that is, the “Support” score, which is simply the number of videos that exemplify that particular implication (we should note that the in-sample validity of the implications does not depend on this score, as these implications hold without exception throughout the sample).

We chose the FCA method for our purposes because other formal methods commonly used for qualitative data analysis are generally considered less expressive, less effective, or inferior in certain aspects compared with FCA (e.g., conventional cross-tabulations of categorical data or typical network analyses of simple co-occurrence patterns).

### Ethics

Ethics approval was not required for this study, as it focused on publicly available YouTube data in accordance with relevant institutional and national guidelines. Videos were considered public if they were accessible to everyone and not password-protected. As a result, individual consent was not required from the authors. However, for the purposes of content analysis and reporting, video identification numbers were created and used to anonymize direct quotes, thereby preserving the anonymity of the video presenters.

## Results

### Characteristics of the videos

Following the sampling procedure described in the methods section, we reviewed 400 videos. Of these, 114 met the inclusion criteria. The total number of views of the included 114 videos was 43,131,651 of which the total time length was 5.4 h (mean duration per video was 10:09 min). The average number of likes was 10,411, and the video on body composition change had the highest number of views (5,237,458).

The presenters were registered dietitians/nutritionists (22.8%), other healthcare professionals (20.2%), athletes (25.4%), fitness and personal trainers (21.9%), other professionals (6.1%), and laymen (3.5%). Overall, 22.8% of the presenters were experts, such as registered dietitians and nutritionists, whereas 77.2% were nonexperts. The videos targeted various athletic groups. The primary focus was on endurance athletes (24.6%) and on a general athletic population without a specific target group (33.3%). Recreational athletes are an important target group (22.8 %). Other categories, such as young athletes (5.3%), strength athletes (1.8%), and martial arts practitioners (2.6%), were less common. Team sports players accounted for 4.4%, whereas special target groups including women and aging athletes accounted for 5.3%.

The same video sample has been used in a previous study, where additional characteristics of the videos are described in detail [18].

## Sport Nutrition Messages on YouTube

**FIGURE 1:**
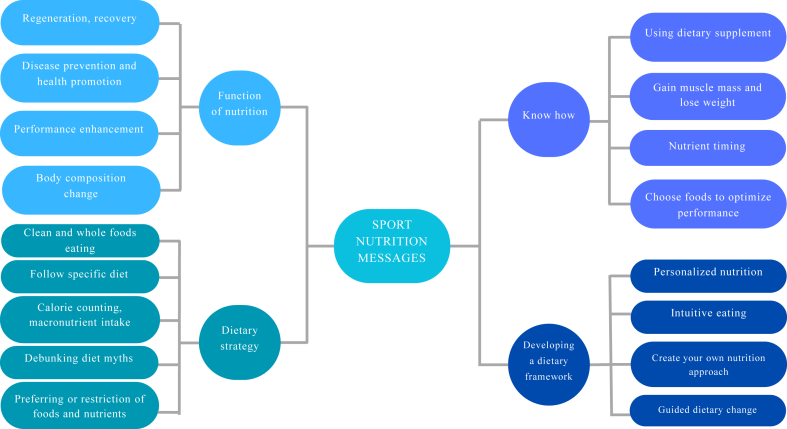
Themes identified from the YouTube videos.

We identified 4 major themes through qualitative content analysis (Figure 1). These include the functions of nutrition, know-how, dietary strategy, and development of a dietary framework for success. There were 4–5 main categories supported for each major theme.

### Function of nutrition in sports

The presenters of these videos attributed particular functions to nutrition and special sports diets. These functions include enhancement of performance, facilitation of recovery, modification of body composition, and improvement of a healthy lifestyle. The most frequently mentioned function of nutrition by the nonexperts was to facilitate recovery after training or competition. The presenters recommended the intake of different nutrients (e.g., protein, carbohydrate, omega-3 fatty acids), dietary supplements (e.g., Branched-Chain Amino Acids (BCAA), whey protein), and foods (e.g., milk, avocado, banana) within a specific timeframe after the training.

“So if you do a lot of endurance training and want to feel more recovered after your sessions, taking a carbohydrate drink with added BCAAs could be a good option.” (Video 16, nonexpert.).

“It’s better to eat protein immediately, less than 2 hours after, to initiate the recovery and muscle reconditioning as soon as possible.” (Video 21, nonexpert).

The role of nutrition in promoting health and preventing diseases is also a key topic. Both experts and nonexperts emphasized the role of healthy nutrition in maintaining a healthy lifestyle, regardless of the goals athletes set for themselves. Nutrition has been introduced as having a primary role in the prevention of noncommunicable diseases (NCDs, e.g., cancer). Although experts introduced general healthy eating guidelines, nonexperts recommended specific diets or consumption of superfoods to maintain health.

“The first part is what's called fundamental nutrition, just eating good, healthy foods for general health, body composition, and your immune system.” (Video 1, Expert).

“Avocado does have an incredible amount of health benefits. This rather insignificant-looking, pear-shaped fruit has such a long list that I'd be here all day if I went through everything. But the few that jumped out to me are: it's high in antioxidants, helps us to digest fat-soluble vitamins, helps balance our cholesterol levels, it's high in fiber, it has plenty of healthy monounsaturated fatty acids, and it's high in potassium.” (Video 61, nonexpert).

The third main category among nutrition functions was performance enhancement. Experts distinguished between “basic” or “fundamental” nutrition, intended to maintain general health, and performance nutrition, which is specifically aimed at enhancing athletic performance. They considered a healthy, balanced diet to be essential, but emphasized that peak performance often requires more targeted nutritional strategies. In contrast, nonexperts typically did not make this distinction and tended to view adequate nutrition in general terms, often attributing performance gains solely to healthy eating.

“The first thing I want to touch on is a concept that I call the fueling performance pyramid. We talk about building this pyramid from the bottom up to have a solid base. The first part is what's called fundamental nutrition…so you've got to be healthy first, then you can put on the performance nutrition piece.” (Video 1, expert).

“So overall good nutrition is going to help your athletic performance because you're using it as your fuel for your workouts.” (Video 34, nonexpert).

## Know-How

The “know-how” theme originated in videos in which the presenters gave practical advice to athletes. In this category, the use of dietary supplements was most common. Nonexperts presented the use of dietary supplements as a general recommendation, suggesting that their use is necessary for every athlete to enhance performance and maintain overall health. The recommended supplements were creatine, protein powder, and caffeine. In addition, the use of dietary supplements has been suggested in cases of nutrient deficiency or special diets (e.g., B12 for vegetarian athletes). In their videos, experts emphasized the responsibility of athletes to use dietary supplements while highlighting the safety concerns and health risks associated with certain supplements.

“There is one supplement that I recommend almost universally: creatine.” (Video 80, nonexpert).

“And the number one stimulant is caffeine! Right. So, caffeine, listen, caffeine is effective.” (Video 26, nonexpert).

“Substances are actually not regulated by the FDA, and they can contain banned substances that can hinder your performance and your health.” (Video 2, expert).

The “know-how” theme also incorporated videos in which nonexpert presenters introduced recommendations for muscle mass gain and body composition management. To achieve these fitness goals, presenters recommend the consumption of certain foods and nutrients. They highlighted the importance of adequate energy and increased protein intake from foods and supplements, the use of vitamin supplements, and the consumption of foods rich in healthy fats, such as avocados. Weight loss is another common topic among nonexperts, and was introduced as a tool for enhancing performance and the goal of body composition change. To achieve these fitness goals, presenters focused on modifying energy balance, decreasing carbohydrate intake, pre- and postworkout nutrient intake, and maintaining special diets. The topic of weight loss was mentioned to a lesser extent in the expert videos; however, when it was addressed, the emphasis was placed on a gradual and sustainable approach.

“..if you reduce the amount of calories that you take in, but you maintain the same amount of training or activity levels, you are going to lose weight.” (Video 81, nonexpert).

“I think I would aim for a slow and gradual weight loss. So certainly no more than one kilo per week.” (Video 17, expert).

Within the “know-how” theme, the videos placed significant emphasis on practical recommendations for the timing of nutrient intake to support training nutrition goals and recovery. The subcategories of nutrient timing in nonexpert videos were nutrition before, during, and after training, and the special workout environment. In the nutrient timing recommendations for pre- and postworkout nutrition, the videos provided practical advice on the optimal timing for athletes to consume specific macronutrients and supplements relative to their training sessions. The recommendations for nutrient intake during workouts were adjusted during the training period. The presenters provided advice on macro- and micronutrient intake and the consumption of certain foods based on their nutrient composition.

“I want to see you have 20 to 40 grams of protein before training and 40 to 60 grams after training. Your 20 to 40 grams can come from a supplement source...” (Video 89, nonexpert).

“A good guideline if you're on a long, intense ride, try to consume some carbohydrate every 20 minutes or so.” (Video 36, nonexpert).

## Diet Strategy

The theme of “nutritional strategies” was identified based on the nutritional approaches presented in the videos. These approaches are characterized by recommendations for food selection, special diets, and energy and macronutrient intake. The first category that appeared in the videos of nonexperts was “pure and whole foods eating” which suggests the consumption of whole, fresh, and minimally processed foods. They pointed out the importance of choosing “natural,” “unprocessed,” “real,” and “high quality” foods. The presenters motivated viewers to avoid genetically modified organism (GMO) foods, artificial sweeteners, and ultraprocessed foods. In parallel, they suggested considering the origin of foods when choosing (e.g., purchasing food from markets instead of groceries or hypermarkets) and consuming home-prepared foods.

“Over the years, I’ve adapted to, basically, just a whole food type of diet, where I just eat real, good, clean food.” (Video 9, nonexpert).

“So, again, some meats or some fish or some eggs will always be better for you because: it's real food, it’s unprocessed.” (Video 56, nonexpert).

As part of their nutritional strategy, athletes commonly receive suggestions for special diets such as plant-based diets, intermittent fasting, or a ketogenic diet. Intermittent fasting and ketogenic diets were recommended to enhance performance within an athlete’s preparation, while explaining how they trigger physiological changes that eventually lead to better performance. A vegetarian diet was introduced as a way to change lifestyles, adding that it can improve performance and maintain the health of athletes. Some of these recommendations were supported by the personal stories of vegetarian athletes. Most of the presenters in these videos did not mention the risks of maintaining special diets. The promotion of these special diets was primarily a characteristic of nonexperts. In contrast, experts offer guidance on diets specifically tailored to medical conditions, such as gluten intolerance or irritable bowel syndrome (IBS).

“So, very clear advantage I think athletically for people, if they just switched over to just eating vegan for performance for sure.” (Video 99, nonexpert).

“For my patient athletes with IBS, we're looking to control a lot of things, sometimes their symptoms are interfering with their practice. We may also be doing dietary modifications to help control symptoms, such as in the case of when we put an athlete with IBS on a low FODMAPs diet.” (Video 5, expert).

Energy intake was emphasized in both expert and nonexpert videos. Their opinion was that adequate energy intake is necessary for optimal performance. However, the nonexperts referred to recommendations for the general population. Moreover, they did not provide recommendations on how to calculate daily energy requirements. Experts discussed adjusting energy intake according to the level of physical activity, sex, and sport type. Both groups provided recommendations on athletes' daily macronutrient intake, expressed daily macronutrient needs as proportions relative to each other, in % of total energy requirement, or adjusted to body weight. Among nonexperts, the most frequently discussed topic was the increased protein intake needs of athletes, which they introduced as a widely accepted dietary principle. Both the experts and nonexperts presented macronutrients based on their dietary sources. When discussing fat intake, nonexperts differentiated between “good” and “bad” types of fats, and according to them, “good” fats/fatty acids are found, for example, in avocado, walnuts or olive oil. Regarding carbohydrates, it was emphasized that their intake correlated with body mass (weight) gain; thus, a decreased intake of carbohydrates was suggested. They also highlighted the role of complex carbohydrates (e.g., whole-grain cereals, fruits, and vegetables) in sports nutrition.

“Your diet should provide enough energy so that you can perform well on the tennis court from a physical standpoint…” (Video 10, nonexpert).

“…there are three macronutrients: protein, fats, and carbs, and your body requires a certain amount of each of these every single day. I can't tell you exactly what your body needs, but a good rough estimate for the average person would be around 35 to 40 percent of your calories coming from carbohydrates, 25 to 30 percent coming from protein sources, and around 30 percent coming from fat.” (Video 34, nonexpert).

“One of the big sources of the confusion is that most of the nutrition messages that go out are targeted to the two-thirds of Americans who are overweight or obese, and athletes who are health conscious and nutrition hungry listen to all these messages that aren't meant for them.” (Video 18, expert).

The nonexperts’ recommendations regarding food groups and consumption were based mainly on their nutrient content, personal beliefs, and nutritional myths. By contrast, experts provided advice on food consumption based on healthy and sports-specific nutritional guidelines, such as the Athlete Eating Guidelines developed by the United States Olympic & Paralympic Committee’s (USOC) sport nutrition team.

“...the problem with dairy is it contains the actual sex hormone of estrogen. So if you ingest a lot of dairy, you're ingesting female sex hormone.” (Video 93, nonexpert).

“So my personal opinion about beetroot juice is that I think it's a potential natural performance enhancer. I believe that it helps.” (Video 22, nonexpert).

“A good way to look at how much I should be eating or how my distribution should be is that we have these great resources from the USOC Sports Nutrition Group, the US Olympic Committee” (Video 1, expert).

## Establishing a Dietary Framework for Long-Term Success

Presenters provided dietary frames for creating dietary strategies that helped reach and maintain personal and sports aims. Intuitive eating was a prominent nutritional approach to achieve long-term success. In the framework of intuitive eating, the most important factors were mentioned: detecting signs of hunger and satiety, a positive relationship with foods, and well-informed decisions about the quantity and quality of foods to be consumed.

“Have a good relationship with your food.” (Video 79, nonexpert).

“If you listen to your body, you'll start to learn. Oh, this is actually thirsty.” (Video 15, nonexpert).

Adjusting nutrition to athletes’ needs and lifestyles is another commonly shared advice. Both experts and nonexperts stressed that there is no universal diet for everyone with different needs and goals. Nutrition plans should be adapted to the specific requirements of athletes; therefore, a personalized diet plan is recommended by the presenters. However, the concept of personalized nutrition varied between the 2 types of presenters. Experts provided guidance based on nutrient intake recommendations tailored to sports, physical activity level, sex, and age. In contrast, nonexperts encouraged athletes to create their nutritional approach through trial-and-error, research, and self-education.

“Every individual is different and we need to address that. So, for example, the exercise intensity and the duration, the type of sport you do, determine much more what you need than your genome.” (Video 42, expert).

“So there is a little trial-and-error in creating the correct hydration strategy. I urge you again to find the method that works for you.” (Video 62, nonexpert).

Additionally, both groups offered general principles for the implementation of nutritional strategies and dietary changes. Some presenters pointed out the importance of diverse eating habits, whereas others suggested step-by-step progress in changing diets. They also explained the importance of rules and habits in establishing dietary habits.

“But I believe if you can find an approach that works for you, that you can stay consistent with, that won't sacrifice your health in the process; I'm all on board for it.” (Video 102, nonexpert).

“..this is a really good time to create habits, and this is what I've been telling all of my clients lately: build habits now that you can sustain later. However, if you stick to the key points, and you make sure you do any changes gradually, consistently, and look after your overall health.” (Video 54, nonexpert).

“The key is about understanding what you need to get and being consistent with it.” (Video 1, expert).

“So when it comes to actually devising your daily meal plans, fueling your body adequately, you wanna think about abundance, variety, and balance.” (Video 58, expert).

## Way of Conveying Messages on Nutritional Information

Table 2 summarizes the various methods and techniques applied to YouTube videos to convey nutritional information. Four themes were identified: language features, content delivery methods, appearance to be connected to the audience, and establishing credibility.

**TABLE 2:** Ways of conveying sports nutrition information in YouTube videos.

Themes for conveying nutrition information	Main categories
Language features	Communication style
	Persuasive language techniques
Content delivery methods	Mode of content delivery
	Style of content delivery
Appearing connected to the audience	Appearing similar to the audience
	Building a community with the audience
Establishing credibility	Citation of different sources of nutrition information
	Inclusion of scientific fact
	Using technical/simple language
	Positioning as experts

### Establishing credibility

Nonexpert presenters supported their dietary claims and sports nutrition recommendations by referencing external sources to establish credibility. These recommendations originate from different sources, including scientific publications and their citations, independent experts, such as sports nutrition experts, research results published by universities, and statements from medical professionals and independent professional organizations. Among experts, nonexperts most frequently cited dietitians to support dietary recommendations. In the videos, presenters introduced snippets from the diets of certain athletes or Olympians or interviewed them about the advantages of the nutrition promoted by the presenter.

“Now, studies have actually suggested that consuming high levels of nitrate can help with athletic performance, especially when it comes to endurance athletes.” (Video 3, nonexperts).

“In regards to the greatest health concern of kidney damage, a 2019 systematic review and meta-analysis analyzing the effects of creatine supplementation on renal function thought that supplementation did not induce kidney damage.” (Video 31, nonexpert).

In addition, a common communication method used by nonexperts is to highlight the print screens of scientific publications, short texts, figures, tables, and titles. However, these snippets, often limited to a single sentence, were presented without proper context or further explanation, leaving it unclear whether the research targeted athletes or whether the findings were relevant to them. References to the sources of these results are usually inadequate to identify original studies. Although presenters referred to scientific results, they often mixed them with their own opinions or made them insignificant compared with their statements based on their own research.

“When it comes to nutrition, I rely heavily on the research that's been published in journals and done by scientists all over the world. But just because something has been discovered in the lab does not mean that it's necessarily gonna carry over into the real world.” (Video 51, nonexpert).

In contrast, experts also referenced scientific studies but focused on research specifically relevant to athletes, providing a context to ensure that the findings were applicable to their audience.

“Research has found that up to 69% of women meet the criteria for one or two components of the Triad, particularly in sports where leanness is commonly encouraged, such as gymnastics or ballet.” (Video 69, expert).

Additionally, nonexperts often employed technical or scientific language to increase their credibility; they often explained metabolic and physiological functions using medical and scientific terms. In contrast, experts used simple language in their videos, and when technical terms were necessary to explain physiological processes, they clarified them to their audience, ensuring better understanding.

“...glycogen reserve replenishment, new capillaries are grown, new mitochondria cell structures are repaired, enzymes are restored to the things that actually do all the work in the body...” (Video 50, nonexpert).

“Glycogen is the main source of energy of the muscles. So after exercise, we need to refuel this glycogen storage.” (Video 21, expert).

In the sports nutrition videos, both experts and nonexperts shared their professional backgrounds, including relevant degrees and specialized training in the field of sports nutrition, to show their authenticity. Furthermore, they shared experiences such as the year-long coaching of sports teams and individual athletes. Nonexpert presenters sometimes introduced their experiences in sports and shared anecdotes from their careers to highlight how their advice may help athletes reach their goals.

“You know, as a good coach, I think a lot of times we have to resort to our own experiences in order to be able to coach from. Pre- and postworkout nutrition is one of the most common questions I've gotten from clients in over 20 years of coaching.” (Video 45, nonexpert).

“I'm a nutritionist and nutrition researcher at Liverpool John Moores University.” (Video 16, expert).

### Language features

One of the themes identified in the analysis of conveying sport nutrition information was the language features the presenters used to communicate with their audiences in the video. Within this theme, 2 main categories were identified: communication style and linguistic techniques used. The videos employed various communication styles, including positive, advisory, argumentative, analytical/explanatory, motivational, accusatory, and dramatic.

Experts tended to use a positive, advisory, and analytical tone, whereas nonexperts applied an argumentative, motivational, and dramatic tone. Many nonexperts adopted an accusatory tone in several videos, with the recurring theme of blaming the food industry for misleading consumers. They claimed that food manufacturers produce and promote products containing hidden substances or ingredients, such as added sugars, making it more difficult for consumers to make healthy choices.

“And I want to show you guys one of the biggest tricks that sport drinks will play when they’re trying to hide some of the things that aren’t so great about their drink.” (Video 29, nonexpert).

The nonexperts delivered nutritional advice using precise and authoritative language. Their messages often implied that consuming specific foods (e.g., superfoods) and nutrients in exact amounts would bring about dramatic, almost magical changes in the body. These claims listed numerous nutrition-related benefits for athletes with little explanation, further reinforcing their assertive tone. Presenters’ confident delivery and decisive tone helped strengthen their perceived roles as professionals or experts.

“What happened after I made the change was just shy of extraordinary. It was literally almost, it was, it was just, it was kind of magical.” (Video 68, nonexpert).

n the persuasion technique main category, the use of rhetorical devices dominated. Nonexperts frequently employed hypophora, in which presenters posed and answered questions. Additionally, they often exaggerate the physiological and performance-enhancing effects of specific dietary recommendations. Dramatic terms, such as “toxic” and “deadly,” were used to stress the consequences of following inadequate diets. Nonexperts also used repetition to emphasize key messages and drive them to the audience. In contrast, experts relied on rhetorical tools such as parallels and similes to make their points more relatable.

“Alcohol is perceived to be a toxin like your processed foods.” (Video 108, nonexpert).

“When we eat meat, it’s dead protein because it's been caught, it's been put in the freezer, it's been cooked. It’s been in the, you know, it has been manipulated so many ways with Human Growth Hormone (HGH) chemical, and it’s a dead protein that is not good. And it's staying in our body for so long.” (Video 68, nonexpert).

“Supplements are the sprinkles on the icing on the cake, and you have to get your cake with your basic nutrition right. If you haven’t got a cake and haven’t iced it, then a packet of sprinkles is no use to you at all.” (Video 4, expert).

### Content delivery methods

Presenters employ various methods to deliver their content. The first category in this topic focused on the modes of content delivery, where presenters used different approaches, such as sharing recipes, displaying screenshots, providing detailed guides, conducting cooking demonstrations, and offering food lists and bullet point summaries.

The second category examines the style of content delivery. Multiple messages were conveyed in the videos, with presenters typically setting the purpose at the beginning, using compelling headlines, and clarifying what viewers expected within the first few sentences. Nonexperts’ recommendations were not limited to nutrition alone; they often included behavioral, lifestyle, and motivational advice. In addition to sharing personal experiences and presenting guest interviews with athletes, the presenters employed various content delivery styles. These include narrative storytelling, educational demonstrations, comparative analyses, and motivational speeches.

One aspect of the content delivery style of nonexperts can be characterized as an emotional and value-based approach. Sports nutrition messages were embedded in content designed to evoke strong emotional responses such as childhood memories, animal welfare, and environmental concerns. This approach was particularly noticeable in videos promoting vegetarian diets, in which food choices were framed as value-driven decisions. This content delivery style encourages the audience to adopt specific diets by appealing to their emotions and values.

“…whole food Plant-Based diet is very sustainable long-term you’re not really eliminating anything except you know the animal kingdom and there's just a tremendous variety of plants that are available…” (Video 94, nonexpert).

Presentation environment plays a significant role in YouTube videos. Nonexpert presenters aim to choose locations that resonate with their target audience such as kitchens, outdoor settings, gyms, and training locations. These choices of environment are often aligned with the content delivery styles employed, enhancing viewer engagement and overall experience. For example, cooking and meal preparation demonstrations are typically filmed in kitchens, whereas workout routines are recorded in gyms or outdoor spaces, creating settings that feel relevant and authentic to viewers. By contrast, expert videos are often recorded in office environments.

### Appearing connected to the audience

Establishing a connection with the audience was a key theme, particularly for nonexpert videos. Two main categories emerged: similarities with the audience and community building with viewers. The first category, similarity with the audience, focuses on presenters’ relatable qualities. They appeared approachable and sincere, expressed support for their audience, and reflected on the attitudes shared by their target viewers. This also extends their physical appearance. Presenters often aim to match the norms of their audiences’ expectations in terms of physique and style. In videos centered on muscle building and weight management, the presenters typically had the type of body that the viewers desired and wore outfits that highlighted their physique.

“We’re going to get up close and personal here, today. You and I. Pull up a chair. Let's spend some time together.” (Video 43, nonexpert).

“…if you guys want to reach out to me, talk to me if you have any questions, I would be happy to help you out any way that I can. I'm on this journey with you.” (Video 66, nonexpert).

In this category, presenters portrayed themselves as similar to their audiences, often highlighting that they were athletes themselves or featured athletes in their videos. The presenters featured individuals who were attractive within the culture of their sport and relatable to their audience. They frequently shared their personal experiences and showcased their diets. Throughout the presentations, the presenters positioned themselves as individuals facing similar life situations and nutritional challenges to their viewers. This approach reassured the audience that the presenters understood their struggles related to nutrition and were familiar with their needs.

“uh every single day, you know, trying to control my weight, trying to control what goes in my body, but I make mistakes too. I don't want to make it seem like I've never had cake or never had or never get any type of urges. I have urges all the time to eat those types of things as well...” (Video 66, nonexpert).

“I started Meat-Free Athlete basically just as a way for me to show by example how I’m doing that and just my journey as a vegan athlete, and what’s working for me, and just showing the success I’m having athletically.” (Video 99, nonexpert).

### Building a community with viewers

The second main category focused on creating a sense of connection with viewers and understanding their needs, particularly for nonexpert videos. Presenters actively monitor and respond to viewers’ comments and questions, and often shape videos based on audience feedback. They build a sense of community by actively interacting with comments and encouraging viewers to subscribe to their channels or buy the products they promote. This active communication strengthens the connection between the presenters and their audiences, leading to an engaged community.

“I did do a video probably about a year ago that was about just gaining weight for athletes, and I still get a lot of engagement, a lot of comments, a lot of people asking questions on that video, so I thought I would go ahead and make a video here that goes more into some specific foods to help you gain weight.” (Video 66, nonexpert).

“Do you take any pre-workout supplements? Let me know which ones in the comments. And if you are curious about my pre-workout rec's, I'll link to some of my favorites in the description below.” (Video 49, nonexpert).

“I am waiting for your questions, comment them in the comment box. I will pick up some good topics and will be discussed in the coming days.” (Video 53, nonexpert).

## The Structure of Associations between the Style of Communication, Video Theme, and Expertise

On the basis of the results of our qualitative analyses (determination of communication methods, sports nutrition themes, and expertise), we used FCA to show the associations among the style of communication, sports nutrition messages, and expertise. We defined expert communication in terms of both methods and key sport nutrition messages, contrasting it with nonexpert communication. We identified 2 sets of schemes.

The first set is of scheme (1), {X & … & Z} ⇒ E1/E0, meaning that the coappearance of communication methods and sports nutrition messages (X and … and Z) implies an expert/nonexpert. Many categories imply experts, and many categories imply nonexperts. Because the latter are mutually exclusive categories (so that if 1 is implied, the other cannot be), we can conclude that the set of communication features is divided along expertise, that is, most expert and nonexpert communication styles are categorically different: FCA shows that nonoverlapping sets of different communication features are definitive of expertise compared with nonexpertise. For example, implication no. 36 in Table 3 shows that if “debunking diet myths” and “inclusion of scientific fact” appear in the video together as features of a dietary message and style of communication, respectively, then the video belongs to an expert (i.e., “E-1” is a necessary feature of the video in the sample). More colloquially, the implication is that the clarification of diet myths appealing to scientific facts implies expertise. Another implication is that if scientific facts and “linking diet to exercise outcomes” are presented together, the video was created by an expert.

**TABLE 3:** Communication features implying an expert (support ≥ 2).

ID	Support	Premises	Implication
9	6	“describing benefits of eating”	E-1
10	6	“recommend expert consultation”	E-1
13	5	“linking diet to exercise outcome” and “personalized nutrition”	E-1
15	5	“linking diet to exercise outcome” and “simple language”	E-1
16	5	“personalized nutrition” and “simple language”	E-1
21	4	“inclusion of scientific fact” and “linking diet to exercise outcome”	E-1
36	3	“debunking diet myths” and “inclusion of scientific fact”	E-1
52	3	“discussing potential risk of supplements” and “sport-specific dietary recommendation”	E-1
84	2	“inclusion of scientific fact” and “personalized nutrition”	E-1
121	2	“linking diet to exercise outcome” and “sport-specific dietary recommendation”	E-1
202	1	“discursive tone” and “linking diet to exercise outcome”	E-1

ID: implication number from the entire implication set. Support: the number of videos that exemplify that particular implication. Premises are features or the conjunction of certain features. E-1: expert.

Table 4 presents the features and coappearance of the 2 features, implying a nonexpert. Many features imply nonexperts; for example, the most supported implications were not distinguishing between fact and opinion or describing diet in the form of a philosophy. Implication no. 27 indicates that when “linking micronutrients to physiological effect” is coupled with “using technical language,” it implies a nonexpert video. Sharing one's story paired with visually appealing content also implies that the video was created by a nonexpert.

**TABLE 4:** Communication features (style) implying a nonexpert (support ≥ 2).

ID	Support	Premises	Implication
1	20	“not distinguishing between fact and opinion”	E-0
2	12	“describing diet in the form of a philosophy”	E-0
3	8	“detailing personal experiences”	E-0
4	8	“focus on food as a means to achieve goals”	E-0
5	8	“linking foods to diseases”	E-0
6	8	“positioning as expert”	E-0
7	7	“support of supplement use”	E-0
8	7	“understanding the audience”	E-0
11	5	“encouraging food restriction”	E-0
12	5	“motivational content”	E-0
14	5	“inclusion of scientific fact” and “recommend focusing on food quality and purity”	E-0
17	4	“encouraging athletes to conduct their own research”	E-0
18	4	“encouraging nutrients restriction”	E-0
19	4	“exaggeration”	E-0
20	4	“discussing foods in terms of macronutrients” and “inclusion of scientific fact”	E-0
22	4	“argumentative tone” and “providing macronutrient intake information”	E-0
23	4	“quoting dietitians”	E-0
24	4	“inclusion of scientific fact” and “recommend hydration strategies”	E-0
25	4	“inducing emotion” and “sharing own story”	E-0
27	4	“linking micronutrients to physiological effect” and “using technical language”	E-0
29	4	“sharing own story” and “visually appealing content”	E-0

ID: implication number from the entire implication set. Support: the number of videos that exemplify that particular implication. Premises are features or the conjunction of certain features. E-0: nonexpert.

The second set is of scheme (2) {E1/E0 & X & … & Z} ⇒ {U & … & Q}, which means that if an expert uses the communication features X and … and Z, it is accompanied by the features U and … and Q (in contrast, it is not accompanied when a nonexpert uses it) in all cases in the sample. When experts engage in a certain communication method, it implies further features (e.g., tone or dietary message) that are distinct from those appearing in the case of nonexperts. For example, when experts debunk diet myths that include scientific facts, it is only for the inclusion of scientific facts that they turn to technical language (Table 5, ID 34 and 56, respectively). Another important distinction is that experts offered practical advice to athletes less frequently than nonexperts. However, when they provided practical advice for dietary change, it was accompanied by a focus on personalized nutrition and delivered using simple language (Implication ID 87).

**TABLE 5:** Expert communication implication for characteristic features (support ≥ 2).

ID	Support	Premises	Implication
46	3	“E-1” and “recommend hydration strategies”	Describing benefits of eating
34	3	“debunking diet myths” and “E-1”	Inclusion of scientific fact
56	3	“E-1” and “using technical language”	Inclusion of scientific fact
35	3	“discussing potential risk of supplements” and “E-1”	Sport-specific dietary recommendation
135	2	“E-1” and “inclusion of scientific fact” and “linking diet to exercise outcome” and “using technical language”	Argumentative tone
119	2	“describing benefits of eating” and “E-1” and “sport-specific dietary recommendation”	Discussing potential risk of supplements
122	2	“E-1” and “reference to dietary recommendation” and “sport-specific dietary recommendation”	Discussing potential risk of supplements
91	2	“E-1” and “providing macronutrient intake information”	Inclusion of scientific fact
69	2	“argumentative tone” and “E-1”	Inclusion of scientific fact and “linking diet to exercise outcome” and “using technical language”
98	2	“E-1” and “personalized nutrition” and “recommend expert consultation”	Linking diet to exercise outcome
117	2	”E-1” and “recommend expert consultation” and “simple language”	Linking diet to exercise outcome
87	2	“E-1” and “practical advice for dietary change”	Personalized nutrition and “simple language”

ID: implication number from the entire implication set. Support: the number of videos that exemplify that particular implication. Premises are features or the conjunction of certain features.

In contrast to experts, nonexperts appeal to scientific facts in a very different setting according to the implication set, for example, together with supporting supplement use and confounding facts and opinions, always delivering these messages with an argumentative tone (Table 6, ID 26 and 45). Implication number 55 reveals that whenever a nonexpert includes motivational content along with showing an attitude of understanding the audience, it is always accompanied by sharing an athlete's nutrition story” (where the scope of “always” is the sample under study).

**TABLE 6:** Nonexpert communication implications for characteristic features (support ≥ 2).

ID	Support	Premises	Implication
26	4	“E-0” and “inclusion of scientific fact” and “support of supplement use”	argumentative tone
45	3	“E-0” and “inclusion of scientific fact” and “not distinguishing between fact and opinion” and “recommend focusing on food quality and purity”	argumentative tone
54	3	“detailing personal experiences” and “E-0” and “support of supplement use”	argumentative tone
55	3	“E-0” and “motivational content” and “understanding the audience”	sharing athletes nutrition story
33	3	“argumentative tone” and “detailing personal experiences” and “E-0”	support of supplement use

ID: implication number from the entire implication set. Support: the number of videos that exemplify that particular implication. Premises are features or the conjunction of certain features.

## Discussion

To our knowledge, this is the first study to explore the main messages of popular sports nutrition videos and examine the various methods used to convey these messages on YouTube. It acknowledges the increasing popularity of such videos as sources of nutrition information and the growing trend of both experts and nonexperts using the platform to share their knowledge. The themes that emerged from the videos reflect their educational nature. Experts primarily focused on theoretical knowledge transfer and general sports nutrition recommendations, whereas nonexperts tended to provide practical advice for athletes. In addition to practical food selection and specific nutrition advice, the videos also addressed the role and use of supplements, sports foods, and superfoods in athletes’ diets.

Mete et al. [22] analyzed popular healthy eating blogs and found that a key common feature was the practical presentation of healthy eating information. Bloggers shared knowledge through practical tips and suggestions that readers could easily incorporate into their daily routines. According to Mete et al. [22], this emphasis on practical content highlights the importance of conveying procedural knowledge—showing how to apply information in everyday life—rather than merely sharing declarative facts. The presence of practical sports nutrition content in the videos, along with their high viewership, suggests that athletes actively seek such material. This presents a unique opportunity for sports nutrition education and health promotion programs to communicate procedural knowledge. However, further research is needed to determine whether procedural knowledge effectively drives behavior change in athletes, particularly in the context of social media.

On the basis of the results of the qualitative analysis, we conducted a FCA, which revealed co-occurrences between communication methods and sports nutrition messages, indicating whether the video was created by an expert or nonexpert. In doing so, we exposed to experts the structural communication features commonly used by nonexperts when conveying nutrition messages. Understanding the needs of athletes emerged as a key factor influencing multiple forms of message delivery in nonexpert videos. Presenters selected communication methods tailored to the preferences and expectations of their target audience, promoting effective engagement. By sharing case studies, personal anecdotes, and insights into their own diets, presenters not only demonstrated their expertise but also fostered a deeper connection with viewers, enhancing the perceived credibility and trustworthiness of the nutritional information. Sharing personal experiences to increase relatability and credibility was also emphasized by Jenkins et al. [46]. Similarly, studies by Mete et al. [22] and Pilgrim et al. [47] underscored the importance of understanding audience preferences and building a sense of similarity between presenter and viewer for effective communication of nutritional messages.

Encouraging viewers to share experiences and ask questions in the comments fosters a strong sense of community. Analyzing these comments could be highly valuable for future research, as it may help identify common misconceptions or knowledge gaps, as well as topics of interest to athletes. Additionally, tracking comments over time could reveal emerging trends in sports nutrition and shifts in athletes’ interests, allowing educational materials to remain timely and relevant.

Nonexpert video presenters sought to emphasize their nutritional advice through various language features, such as an authoritative and argumentative tone. Although some of the nutrition messages shared were scientifically sound and included medical and scientific terms, as well as physiological mechanisms, these were usually inaccurate and not evidence-based. Chan et al. [21] found that nonexpert bloggers often used technical language to establish credibility. However, this approach is unlikely to enhance athletes’ nutritional literacy, as a high level of health literacy is essential to interpret and apply such information. One of the most common ways to establish credibility was by including scientific facts and citing journal articles in the videos. This practice is supported by previous research [21,23,24], which found that authors of sports magazines and healthy eating blogs frequently cited scientific studies to improve their professional image. However, in this study, scientific findings were often interwoven with the presenters’ personal opinions, making it difficult for viewers to distinguish evidence-based information from subjective viewpoints. The scientific studies cited typically did not target athlete populations. In many cases, the information was selectively presented, taken out of context, or based on incomplete data. Presenters tended to emphasize only specific scientific findings that supported their claims. Our findings align with those of Cook et al. [23] and Righton et al. [24], who found that dietary advice in magazines frequently relied on pseudoscientific discourse.

The credibility of nutrition content and the level of nutritional literacy of nonexperts or presenters without recognized qualifications can be questionable. Black et al. [48] found that healthcare professionals were perceived as more credible than nonhealthcare professionals on Instagram. The authors emphasized that followers who rely on nutrition content shared by nonhealthcare professionals are potentially exposed to misleading information. Furthermore, food influencers—who often shape public perceptions of nutrition—may not consistently promote food literacy in their communications [49].

However, Moreno et al. [50] note that influencers in physical activity and sport have taken on a critical role in promoting health and well-being through digital networks such as Instagram. The role of social media influencers in public health communication is gaining increasing attention. In Finland, the Prime Minister’s Office launched a communication campaign during the COVID-19 pandemic that enlisted influencers to convey official guidelines. The findings showed that influencers helped shape social norms, a key strategic goal in public health communication [25]. Rogers et al. [16] found that the message delivery methods used by influencers can help build trust in public health messages related to food consumption and nutrition. Given the popularity of the analyzed videos, communicating evidence-based sports nutrition information through influencers may be effective among some athletes. However, further research is needed to fully explore the potential impacts and applicability of this approach in sports nutrition.

Our findings underscore the low level of nutritional literacy among some presenters, as their recommendations to include or exclude certain foods often relied on myths and personal beliefs rather than evidence. Two common misconceptions appeared frequently in the videos—both prevalent among the general population and some athletes: first, the belief that consuming protein in amounts exceeding evidence-based recommendations is necessary for muscle development and performance enhancement, often accompanied by an emphasis on protein supplement use; and second, the belief in a direct link between carbohydrate consumption and weight gain. These findings are consistent with those of Cook et al. [23] and Righton et al. [24].

Identifying these misconceptions presents an opportunity for sports nutrition professionals to engage with athletes, build trust, debunk myths, and highlight the importance of collaboration between experts and nonexperts. In one-quarter of the videos, the presenter was an athlete, and another 13 videos featured interviews with athletes or presented sports nutrition case studies. These videos emphasized sharing knowledge and personal experiences to assist other athletes in navigating common nutrition challenges and improving performance. This indicates that athletes are eager to give and receive nutrition advice from peers, whom they view as credible sources due to their firsthand experience. Athletes often rely on their own experiences and make nutrition decisions through a trial-and-error approach, particularly in competitive sports [51]. This underscores the critical role of trust in effectively communicating nutrition recommendations to athletes. They tend to trust medical information that is validated through practice and originates within their sport-specific networks [52]. In light of this, experts may benefit from illustrating nutrition topics through athlete case studies or featuring athletes in educational content. This approach may enhance the credibility of experts and help foster trust between athletes and professionals.

### Study limitations and strength

One limitation of our study is that we focused exclusively on YouTube videos, although other social media platforms may also serve as important sources of nutritional information for athletes. Additionally, analyses of viewer reactions such as comments were extended beyond the scope of this study. Such feedback can offer athletes valuable insights. Another limitation is that we were not always able to verify the professional credentials of the content presenters. In these cases, we assessed their expertise based on the quality of the content they provided.

Despite these limitations, our study provides valuable insights into how sports nutrition messages and communication strategies are conveyed on YouTube. Our findings contribute to the development of best practices for effectively communicating sports nutrition information. One of the strengths of this study is its large sample size, with 114 YouTube videos analyzed. This extensive analysis provides a comprehensive overview of key sports nutrition topics and highlights the diverse target audiences these videos are intended to reach. We based our approach on a qualitative analysis, and the findings were cross-verified using FCA, allowing for triangulation. Triangulation implies that we employed multiple methods to validate our findings, thereby enhancing the credibility and reliability of the results.

In conclusion, the qualitative analysis of 114 sports nutrition YouTube videos identified 4 key themes. One theme addressed the fundamental functions of nutrition, whereas another focused on practical dietary recommendations aimed at enhancing performance. The nutritional strategies theme provided advice on food selection, special diets, and energy and macronutrient intake. Lastly, the theme of establishing a dietary framework focused on building and maintaining a long-term dietary plan to support success in both sports and nutrition.

The analysis also revealed distinct differences in communication styles between experts and nonexperts. Experts tended to deliver evidence-based information, offering dietary advice grounded in nutritional guidelines and official recommendations for athletes. However, these often lacked the engaging and relatable qualities seen in nonexpert videos, which helped attract larger audiences. Nonexperts frequently relied on external sources (e.g., scientific studies and dietitians) and used technical language to establish trust and credibility. They employed persuasive language techniques to influence dietary choices and utilized various content delivery modes and styles, such as engaging narratives and personal experiences. By appearing similar to their audience and fostering a sense of community, nonexperts created a stronger connection with viewers.

These findings highlight the importance of building trust and credibility when delivering sports nutrition information to athletes. Improved digital education strategies are necessary for expert content creators to ensure athletes receive reliable and evidence-based advice. Encouraging collaboration between sports nutrition professionals and nonexperts could enhance the quality and credibility of online content, promote effective nutrition strategies, improve athletes’ nutritional practices, and help prevent the spread of misinformation. Further research is needed to assess the long-term impact of these communication strategies on athletes’ dietary behaviors in the digital age.

## Author contributions

The authors’ responsibilities were as follows – AK, SS: designed the research; AK, OT, SS: participated in data analyses; ÁT, BU-P, ZL: participated in the interpretation of the data, AK: wrote the original draft; OT, ÁT, BU-P, ZL, SS: reviewed and edited the paper; AK: primary responsibility for the final content; and all authors: read and approved the final manuscript.

## Data availability

Data described in the manuscript, code book, and analytic code will be made available on request.

## Declaration of generative AI and AI-assisted technologies in the writing process

During the preparation of this work the authors used Grammarly to improve language and readability. After using this tool, the authors reviewed and edited the content as needed and take full responsibility for the content of the publication.

## Funding

The authors reported no funding received for this study.

## Conflict of interest

The authors report no conflicts of interest.

## References

[1] Pollard C.M., Pulker C.E., Meng X., Kerr D.A., Scott J.A. (2015). Who uses the internet as a source of nutrition and dietary information? An Australian population perspective. J. Med. Internet Res..

[2] Mayfield B.J. (2020). Communicating Nutrition: The Authoritative Guide, Academy of Nutrition and Dietetics.

[3] Bourke B.E.P., Baker D.F., Braakhuis A.J. (2018). Social media as a nutrition resource for athletes: a cross sectional survey. Int. J. Sport Nutr. Exerc. Metab..

[4] Trakman G.L., Forsyth A., Hoye R., Belski R. (2019). Australian team sports athletes prefer dietitians, the internet and nutritionists for sports nutrition information. Nutr. Diet..

[5] Alexa (2023). https://www.alexa.com/topsites.

[6] Arthurs J., Drakopoulou S., Gandini A. (2018). Researching YouTube. Convergence.

[7] Haslam K., Doucette H., Hachey S., MacCallum T., Zwicker D., Smith-Brilliant M. (2019). YouTube videos as health decision aids for the public: an integrative review, Can. J. Dent. Hyg..

[8] Szmuda T., Syed M.T., Singh A., Ali S., Özdemir C., Słoniewski P. (2020). YouTube as a source of patient information for coronavirus disease (COVID-19): a content-quality and audience engagement analysis. Rev. Med. Virol..

[9] Stellefson M., Chaney B., Ochipa K., Chaney D., Haider Z., Hanik B. (2014). YouTube as a source of chronic obstructive pulmonary disease patient education: a social media content analysis. Chron. Respir. Dis..

[10] Keelan J., Pavri-Garcia V., Tomlinson G. (2007). YouTube as a source of information on immunization: a content analysis. JAMA.

[11] Erdem M.N., Karaca S. (2018). Evaluating the accuracy and quality of the information in Kyphosis videos shared on YouTube. Spine (Phila Pa 1976).

[12] Erdem H., Sisik A. (2018). The reliability of bariatric surgery videos in YouTube platform. Obes. Surg..

[13] Ferhatoglu M.F., Kartal A., Ekici U., Gurkan A. (2019). Evaluation of the reliability, utility, and quality of the information in sleeve gastrectomy videos shared on open access video sharing platform YouTube. Obes. Surg..

[14] D’Souza R.S., D’Souza S., Strand N., Anderson A., Vogt M.N.P., Olatoye O. (2020). YouTube as a source of medical information on the novel coronavirus 2019 disease (COVID-19) pandemic. Glob. Public Health..

[15] Dumas A.-A., Lapointe A., Desroches S. (2018). Users, uses, and effects of social media in dietetic practice: scoping review of the quantitative and qualitative evidence. J. Med. Internet Res..

[16] Rogers A., Wilkinson S., Downie O., Truby H. (2022). Communication of nutrition information by influencers on social media: a scoping review, Health Promot. J. Austr..

[17] Langford A., Loeb S. (2019). Perceived patient-provider communication quality and sociodemographic factors associated with watching health-related videos on YouTube: a cross-sectional analysis. J. Med. Internet Res..

[18] Kiss A., Soós S., Temesi Á., Unger-Plasek B., Lakner Z., Tompa O. (2023). Evaluation of the reliability and educational quality of YouTube™ videos on sport nutrition topics. J. Int. Soc. Sports Nutr..

[19] Diekman C., Ryan C.D., Oliver T.L. (2023). Misinformation and disinformation in food science and nutrition: impact on practice. J. Nutr..

[20] Tam R., Beck K.L., Manore M.M., Gifford J., Flood V.M., O’Connor H. (2019). Effectiveness of education interventions designed to improve nutrition knowledge in athletes: a systematic review. Sports Med.

[21] Chan T., Drake T., Vollmer R.L. (2020). A qualitative research study comparing nutrition advice communicated by registered Dietitian and non-Registered Dietitian bloggers. J. Community Healthc..

[22] Mete R., Curlewis J., Shield A., Murray K., Bacon R., Kellett J. (2019). Reframing healthy food choices: a content analysis of Australian healthy eating blogs. BMC Public Health.

[23] Cook T.M., Russell J.M., Barker M.E. (2014). Dietary advice for muscularity, leanness and weight control in Men’s Health magazine: a content analysis. BMC Public Health.

[24] Righton O., Egan P., Russell J.M., Cook T.M., Barker M.E. (2017). Dietary advice for improving cardiovascular health in UK running magazines: a content analysis. Nutr. Food Sci.

[25] Pöyry E., Reinikainen H., Luoma-Aho V. (2022). The role of social media influencers in public health communication: case COVID-19 pandemic. Int. J. Strateg. Commun..

[26] Creswell J.W., Clark V.L.P. (2017).

[27] Fetters M.D., Curry L.A., Creswell J.W. (2013). Achieving integration in mixed methods designs – principles and practices. Health Services Res.

[28] Kahlke R.M. (2014). Generic qualitative approaches: pitfalls and benefits of methodological mixology. Int. J. Qual. Methods.

[29] Percy W.H., Kostere K., Kostere S. (2015). Generic qualitative research in psychology. Qual. Rep..

[30] Sandelowski M. (2000). Whatever happened to qualitative description?. Res. Nurs. Health..

[31] Ori E.M., McHugh T.L.F., Berry T.R. (2022). A qualitative exploration of exercise blog believability among emerging adult women. Qual. Res. Sport Exerc. Health.

[32] (2023). YouTube.

[33] Fink H.H., Mikesky A.E. (2021).

[34] (2023). trends.google.com.

[35] (2006). iProspect Search Engine User Behavior Study.

[36] Beautemps J., Bresges A. (2021). What comprises a successful educational science YouTube video? A five-thousand user survey on viewing behaviors and self-perceived importance of various variables controlled by content creators. Front. Commun..

[37] (2023). YouTube Data API.

[38] Hamad E.O., Savundranayagam M.Y., Holmes J.D., Kinsella E.A., Johnson A.M. (2016). Toward a mixed-methods research approach to content analysis in the digital age: the combined content-analysis model and its applications to health care twitter feeds. J. Med Internet Res..

[39] Elo S., Kyngäs H. (2008). The qualitative content analysis process. J. Adv. Nurs..

[40] (2023). ATLAS.ti Scientific Software Development GmbH. ATLAS.ti Mac. V. 23.2.1. ATLAS.ti Scientific Software Development GmbH.

[41] Cho J.Y., Lee E.H. (2014). Reducing confusion about grounded theory and qualitative content analysis: similarities and differences. Qual. Rep..

[42] Poelmans J., Ignatov D.I., Kuznetsov S.O., Dedene G. (2013). Formal concept analysis in knowledge processing: a survey on applications. Expert Syst. Appl..

[43] Wille R. (2006). Int. Conf. Concept Lattices Appl..

[44] Yevtushenko S.A. (2000). Proc. 7th Natl. Conf. Artif. Intellig. KII-2000, Russia.

[45] R Core Team, R (2021). https://www.R-project.org/.

[46] Jenkins E.L., Ilicic J., Molenaar A., Chin S., McCaffrey T.A. (2020). Strategies to improve health communication: can health professionals be heroes?. Nutrients.

[47] Pilgrim K., Bohnet-Joschko S. (2019). Selling health and happiness how influencers communicate on Instagram about dieting and exercise: mixed methods research. BMC Public Health.

[48] Black A., Fernandez M.A., Desroches S., Raine K.D. (2019). Education matters: certified health professionals have higher credibility than non health professionals on Instagram, Alberta Acad. Rev..

[49] Teunissen L., Van Royen K., Goemans I., Verhaegen J., Pabian S., De Backer C. (2024). How are food influencers’ recipes promoting food literacy? Investigating nutritional content, food literacy and communication techniques in Instagram recipes. Br. Food J..

[50] Moreno D.R., Quintana J.G., Riaño E.R. (2023). Impact and engagement of sport & fitness influencers: a challenge for health education media literacy. Online J. Commun. Media Technol..

[51] Robins A., Hetherington M.M. (2005). A comparison of pre-competition eating patterns in a group of non-elite triathletes. Int. J. Sport Nutr. Exerc. Metab..

[52] Gerbing K.-K., Thiel A. (2016). Handling of medical knowledge in sport: Athletes’ medical opinions, information seeking behaviours and knowledge sources. Eur. J. Sport Sci..

