# Supplementary material for: Conveying Sport Nutrition Information in YouTube Videos: A Qualitative Content Analysis of Dietary Advice and Ways of Communication
Source: Curr Dev Nutr. 2025 Aug 6;9(9):107525. doi: 10.1016/j.cdnut.2025.107525 (PMC12446209; doi:10.1016/j.cdnut.2025.107525)
Supplement: Multimedia component 2 [file mmc2.docx]

*Supplementary File 1*

Formal concepts and implications

A formal concept lattice, derived from the incidence matrix of videos (as objects of analysis) and features (as codes), is known as a "formal context." The most effective way to analyze this lattice is through implications. These implications, or implication sets, reveal the knowledge embedded within the network of formal concepts. It’s important to note that the complete set of implications (deductive inferences) is algorithmically determined based on the structure of the concept lattice, specifically through the relationships between concepts and subconcepts. In this file, we demonstrate a selected exemplary implication set as a subnetwork of the full concept lattice built from our sample to explain the relationship between formal concepts and implications in more detail, highlighting those concepts and linkages in each case that correspond to a particular implication within this set.

The subnetwork of our demonstrative implication set is shown in Supplementary Figure 1. Node coloring reflects some methodological conventions: (1) a half-blue circle stands for a concept with a distinctive feature (code) in its definition: i.e. a feature that is not yet present in its superconcepts or antecedents in the network (“own intent”); (2) a half-black circle stands for a concept that “cuts out” a subset of objects (videos), i.e. videos that share the definitive features of the concept, but no other features of the context (“own objects or extent”). These two (1 and 2) can both be true for a concept, hence the appearance of half-blue, half-black ones. Conversely, some concepts do not bear any of these properties,–no own intent or objects–represented with empty circles.

Regarding the relationship between the network of formal concepts and implications, consider our first example, implication no. 36 from the entire implication set:

(36) {"debunking diet myths" & "inclusion of scientific fact"} ⇒ E-1,

Where “E-1” codes for “Expert”. The implication reads as follows: *if* “debunking diet myths” and “inclusion of scientific fact” appear in the video together as features of theme and style, respectively, then the video belongs to an expert (i.e. “E-1” is a necessary feature of the video in the sample). More colloquially, the implication shows that clarification on diet myths appealing to scientific facts implies an expert. The if-part of the implication on the left-hand side of the arrow is called the *premises*, which, in terms of logic, is the conjunction of certain features. The then-part on the right-hand side is called the *implication*, which is an individual feature (but both sides can contain conjuntions, i.e., the co-appearance of varioius features).

The implication described above is calculated from the concept lattice – Supplementary Figure 2A shows the corresponding subnetwork of concepts. The relevant substructure is highlighted in blue. The implication itself is represented in the terminal node in this subnetwork at the bottom of the highlighted part (hereafter “focal concept”), which is the formal concept subsumed under all the others contributing to the implication (a white circle in this case). Recall that the links between concepts represent subconcept-superconcept relations: hence, the features (intent) of these more general superconcepts, “E1”, “debunking…” and …scientific fact” are all being inherited by the

focal concept, the existence of which shows that this association of features is determinate in the sample: if one is present in a video, the other two will also be there; we can call this a stable configuration of features. Moreover, the network structure also shows that, beyond association, which features imply the presence of the others: when “debunking diet myths” is coupled with “inclusion of scientific fact” – that is, in the focal concept – it is through a concept where “inclusion…” is coupled with “E-1”. In other words, one of the parent concepts of the focal concept is subsumed under “E-1” as a separate concept – therefore, the presence of expertise (E-1) can be deductively implied from the other two (again, associated within the focal concept).

This scheme of reasoning also applies to the other three examples we selected from the full implication set. The examples are chosen to represent the schemes that will be of relevance in the systematic analysis of the results. In particular, the implication

(27) {"linking micronutrients to physiological effect" & "using technical language"}⇒ E-0

is an example of the co-appearance of two features implying a non-expert (Supplementary Figure 2B). Equally instructive is an implication of the form

(55) "E-0" & "motivational content" & "understanding the audience" ⇒ sharing athletes nutrition story

where the expertise code is among the premises (Supplementary Figure 2C). This reads as “whenever a non-expert includes motivational content along with showing an attitude of understanding the audience, it is always accompanied by sharing an athlete's nutrition story” (where the scope of “always” is the sample under study).

| 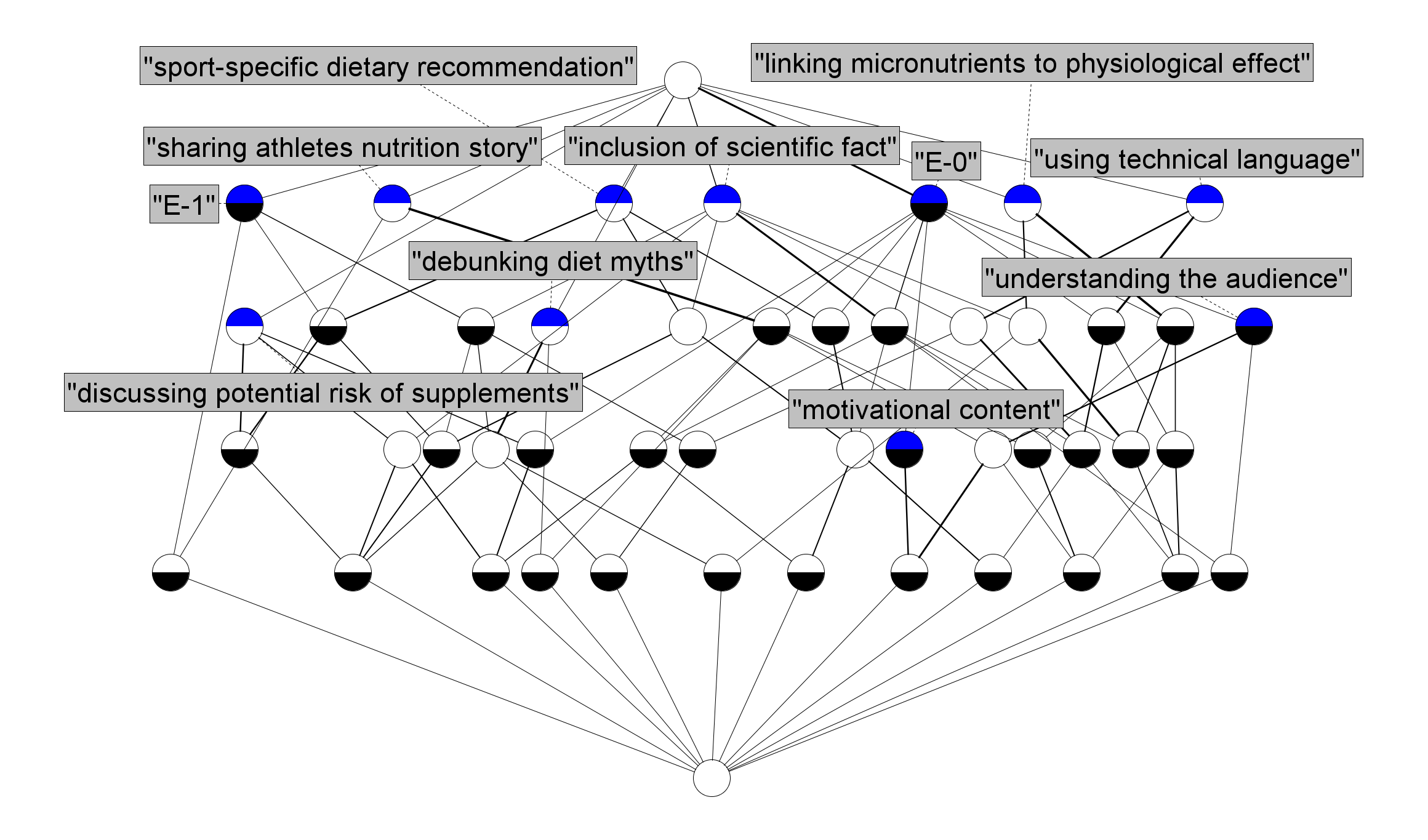 |
| --- |

Supplementary Figure 1. The (partial) concept lattice behind the four example implications (no. 36, 27, 35, 55).

| 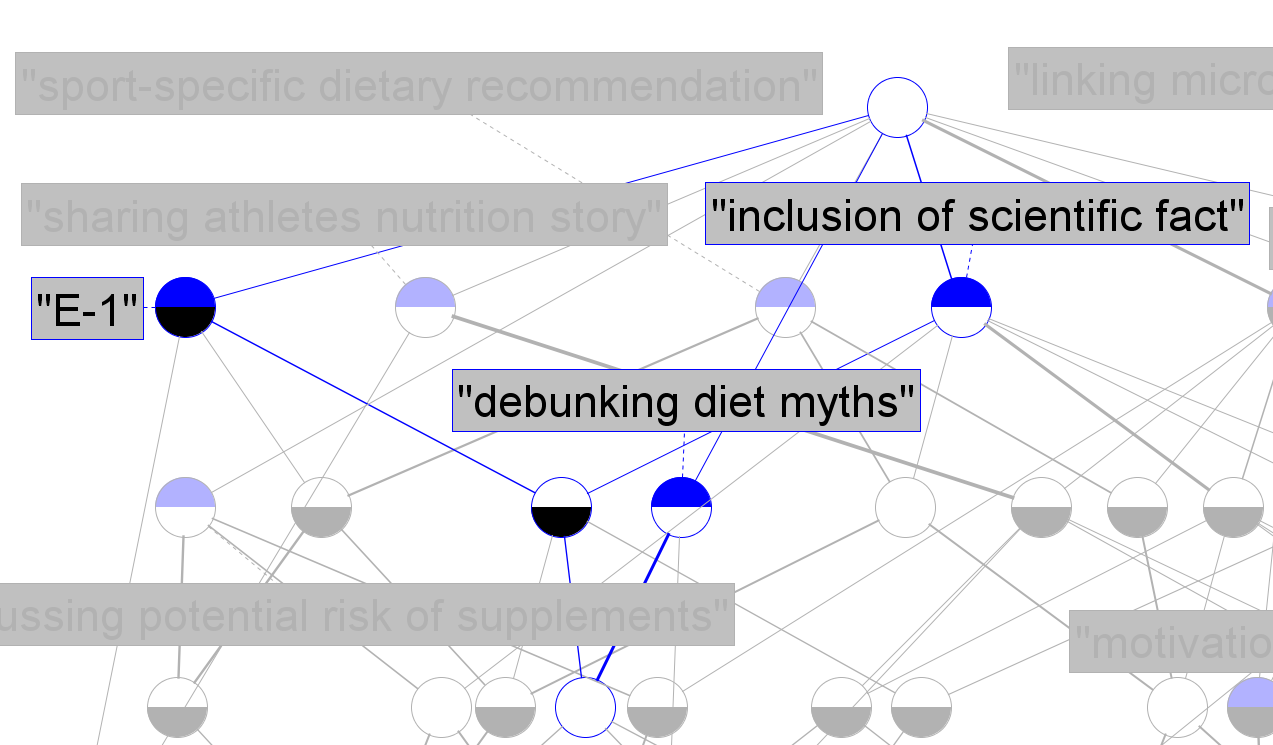  2A | 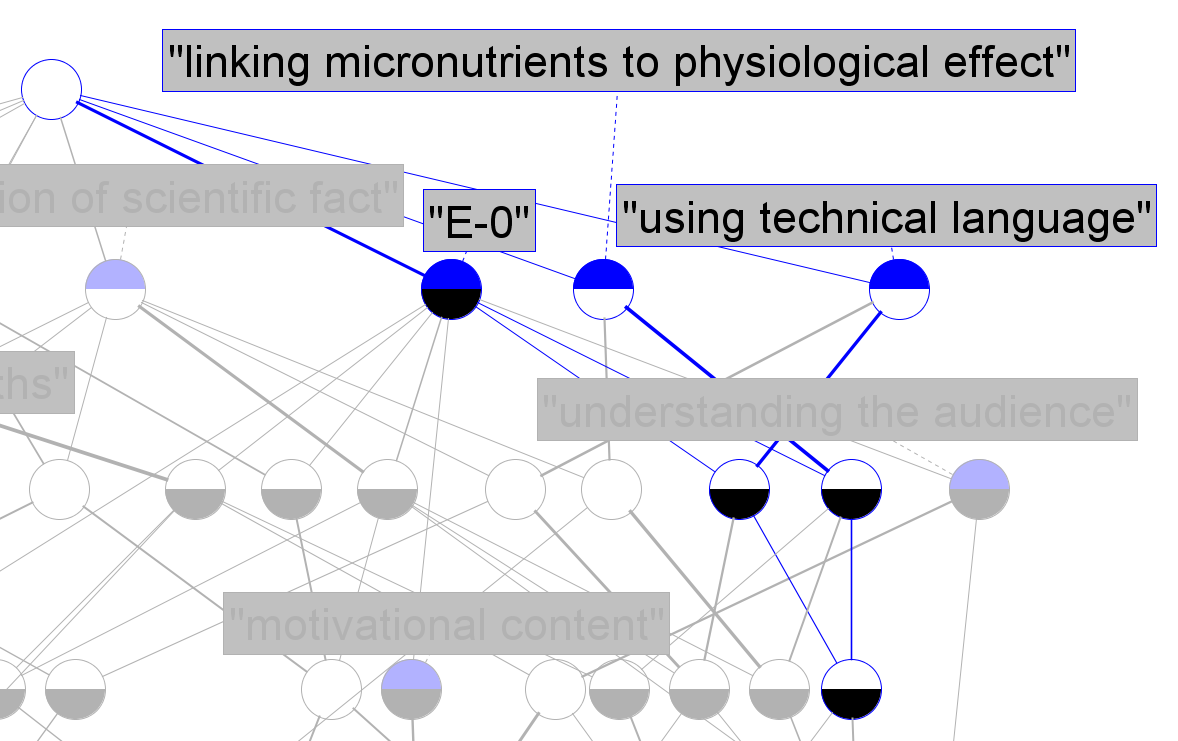  2B |
| --- | --- |
| 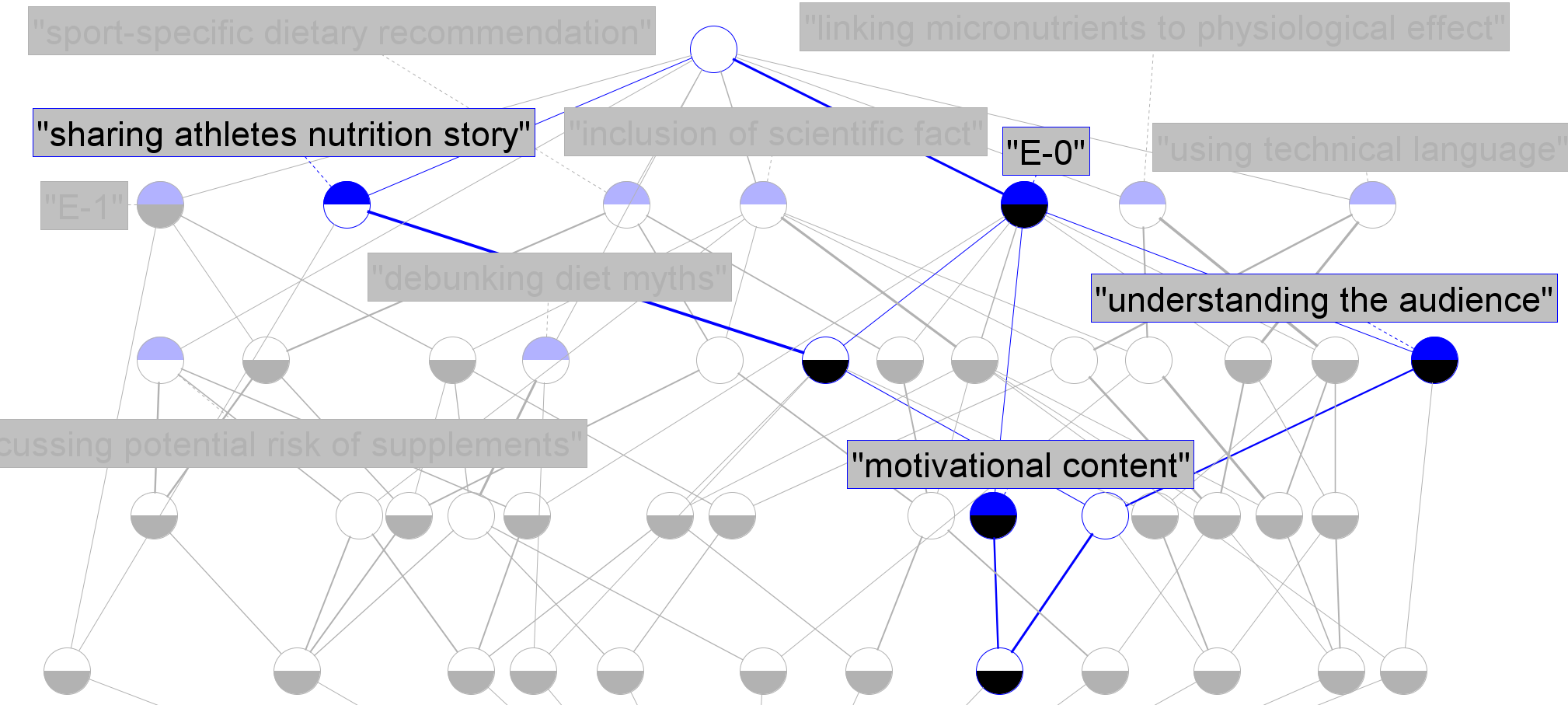2C | |

Supplementary Figure 2. The subgraphs of individual example implications (highlighted in blue). 2A: implication no. 36. 2B: implication no. 27. 2C: implication no. 55.
